# Challenging the N-Heuristic: Effect size, not sample size, predicts the replicability of psychological science

**DOI:** 10.1371/journal.pone.0306911

**Published:** 2024-08-23

**Authors:** Xingyu Li, Jiting Liu, Weijia Gao, Geoffrey L. Cohen

**Affiliations:** 1 Graduate School of Education, Stanford University, Stanford, California, United States of America; 2 School of Humanities and Social Science, The Chinese University of Hong Kong, Shenzhen, Shenzhen, China; 3 Teachers College, Columbia University, New York, New York, United States of America; 4 Department of Psychology, Stanford University, Stanford, California, United States of America; Fundación Universitaria del Área Andina, COLOMBIA

## Abstract

Large sample size (N) is seen as a key criterion in judging the replicability of psychological research, a phenomenon we refer to as the N-Heuristic. This heuristic has led to the incentivization of fast, online, non-behavioral studies—to the potential detriment of psychological science. While large N should in principle increase statistical power and thus the replicability of effects, in practice it may not. Large-N studies may have other attributes that undercut their power or validity. Consolidating data from all systematic, large-scale attempts at replication (N = 307 original-replication study pairs), we find that the original study’s sample size did not predict its likelihood of being replicated (*r*_s_ = -0.02, *p* = 0.741), even with study design and research area controlled. By contrast, effect size emerged as a substantial predictor (*r*_*s*_ = 0.21, *p* < 0.001), which held regardless of the study’s sample size. N may be a poor predictor of replicability because studies with larger N investigated smaller effects (*r*_s_ = -0.49, *p* < 0.001). Contrary to these results, a survey of 215 professional psychologists, presenting them with a comprehensive list of methodological criteria, found sample size to be rated as the most important criterion in judging a study’s replicability. Our findings strike a cautionary note with respect to the prioritization of large N in judging the replicability of psychological science.

## Introduction

Over the last decade, psychological researchers have grown increasingly concerned about the replicability of study findings [1–6]. A key question for editors, reviewers, and funders alike is how to identify the scientific findings most likely to replicate, as well as what features of research to incentivize to ensure more replicable research. One criterion that has been implicitly as well as explicitly emphasized is large sample size [1–3]. For example, one of the field’s top journals, the *Journal of Personality and Social Psychology*, published editorials for three consecutive years, to emphasize the need for large sample sizes [4–6]. More general-interest journals, such as *Nature Human Behaviour*, listed sample size as a key criterion in judging the quality of research studies [7]. A flagship paper on replicability, cited over 11,000 times, posits the following as a “first corollary”: “The smaller the studies conducted in a scientific field, the less likely the research findings are to be true” [8]. Another well-cited paper proposed ranking psychological journals based on the median size of their published studies [9]. Both papers in effect assert that sample size should predict research replicability.

It has also become common practice for journal editors and reviewers to reject manuscripts based on small samples because the studies are believed to be underpowered, even when the obtained effect size is large [4, 7]. Indeed, psychological findings from older studies that used small sample sizes have come under scrutiny, even when they were obtained in rigorous experiments with large effect sizes on behavioral outcomes. Indeed, a large effect size in a small study is seen as a basis for suspicion [10]. Of course, scholars point out the value of other research criteria, and acknowledge that statistical power is not based on N alone [9]. Nevertheless, the overwhelming focus of recommendations and journal policies has been on large N. We refer to this field-wide focus on sample size as the N-Heuristic [4–7].

Of course, larger sample size increases statistical power and, ceteris paribus (all other conditions remaining the same), should increase power and thus replicability. However, whether “ceteris paribus” applies is a key issue. When researchers choose to conduct large-sample studies, they may make other decisions that undermine the replicability of their research. For example, researchers may run large-sample studies but, as a result, put fewer quality control practices in place to ensure that the experimental manipulation was experienced as intended and that the influence of extraneous variables was minimized. They may run studies online, where it is relatively easier to acquire a large N, rather than in the lab, where they would be better able to deliver high-impact manipulations with clear effects rather than light-touch ones with variable results [10–13]. Consistent with this possibility, in 2018, 50% of studies in top journals were conducted online, compared with 3% and 20% in 2005 and 2010, respectively [14].

**Fig 1:**
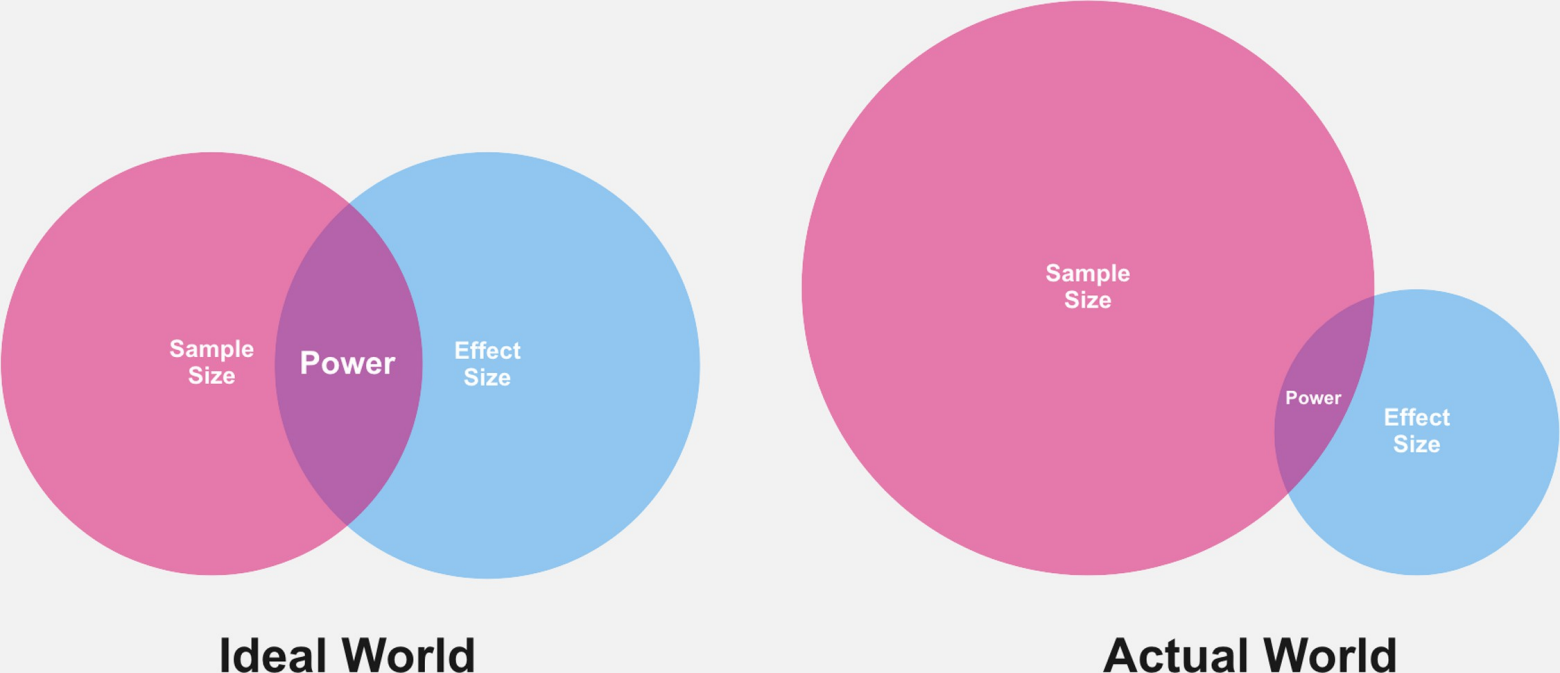
The relationship between sample size, effect size, and statistical power in theory and practice. (Left) In an ideal world, sample size and effect size independently contribute to statistical power; (Right) In an actual world, sample size and effect size are often entangled. Increased sample sizes tend to couple with decreased effect sizes, resulting in less statistical power.

Any one of these decisions will tend to lessen effect size—which, for an experiment, can be defined by the difference between treatment and control conditions, divided by the standard deviation. High-impact manipulations increase the numerator, and experimental and statistical controls that limit the influence of “noise” decrease the denominator. Because effect size contributes to statistical power, incentivizing large N may paradoxically lead to no change in statistical power—or even a decrease in it (see Fig 1). Interestingly, most arguments for large-sample studies are based on simulations and thought experiments that assume that sample size is independent of other research attributes, i.e., that assume “ceteris paribus” [8, 13]. In fact, we do not know the degree of confounding among the multiple predictors of research replicability, or the degree to which each predicts replicability.

The answer to these questions have practical consequences beyond how to ensure statistical power. The N-Heuristic will incentivize certain kinds of research over others, to the detriment of psychological science. For example, although sample sizes have increased in social psychology over the past 15 years, the resulting move to online platforms means that 68% of the studies in top journals now have no behavioral measure of any kind, compared with 39% in 2001 and 46% in 2009 [14]. Roughly a third of studies last fewer than 15 minutes. Self-report measures and short studies are not intrinsically problematic. What is problematic is when a field skews so far in these ways that other kinds of research are crowded out. Because of these factors, some scholars have declared a “relevance crisis” and a “practicality crisis” [15, 16]. The field’s emphasis on large N may lead investigators to eschew (1) research on hard-to-reach populations, studies on groups rather than individuals, (2) labor-intensive laboratory studies, with their rigorous controls, offer a structured approach to approximating real-world phenomena, in contrast to online studies, and (3) longitudinal studies that zero in on a few individuals over relatively longer stretches of time.

Critically, our goal is not to test whether large N *causes* greater replicability. Clearly, for a given study, larger N will increase statistical power and thus the likelihood of identifying a true effect. Our goal is *diagnostic*: Is large N a useful diagnostic for assessing the likely replicability of a research study? In this way, our research is akin to an epidemiological study. An epidemiologist might be interested in a problem, such as suicide, and look for correlates of the problem (e.g., living in urban vs. rural regions). If living in a rural rather than an urban setting predicted higher rates of suicide, this would provide useful information regarding where the problem tends to occur—and where resources and attention should be allocated. In psychological science, the problem under consideration is a perceived lack of replicability; the analogous question is what kinds of studies are the ones to worry about. The prevailing doctrine states that it is the small N studies. Indeed, there are many reasons why small N would be expected to predict less replicability, as noted. Editors and reviewers may have good reason for trusting large N studies more than small N studies. But if it turns out that N does not predict replicability—and that other research attributes do—they might consider using alternative diagnostics in the publication and review process than the ones that now prevail. Put differently, our concern is whether large N is a useful practical *heuristic* for judging the replicability of research in psychological science. We compiled the body of psychological studies over the past 87 years that subjected to rigorous replication attempts and then correlated the size of their original sample with whether the effect they reported was later replicated. Of course, these studies differ in a variety of ways, but our key concern is whether or not one of the ways in which they differ—sample size—predicted replicability—*and* whether other methodological attributes might do a better job at predicting replicability. We control for obvious confounds (e.g., within- versus between-subjects design, the subarea of psychology in which the study was conducted).

## Method

The past decade has yielded many large-scale, international replication projects that now provide abundant data for empirically assessing the predictors of replicable research, see also Open Science Collaboration [17], Yang and colleagues [18], and Altmejd and colleagues [19], and Stanley and Colleagues [20]. Although these replication attempts are not representative of all research studies, they amount to the best empirical data available to assess the predictors of replicable research. These replication attempts also have virtues: Because they were organized by a central organizational body, they followed standardized protocols, including an attempt to obtain original experimental procedures, to pre-specify analysis plans, and to determine sample size based on a power analysis rather than an arbitrary cut-off point. Within a given project, the replication attempts were conducted at roughly the same time. Thus, some key factors that might account for a failure to replicate or contribute to noise (e.g., inadequate power), were controlled. Moreover, while the sample of studies here is likely to be unrepresentative of the larger body of psychological research, an alternative explanation for obtained findings would need to posit why and how a different sampling method (e.g., a representative sampling of all psychological research) would identify different factors predicting replicability. Despite the studies subjected to replication efforts being inherently unrepresentative of all psychological studies, starting the empirical meta-analysis now—without waiting for an ideal, representative sample—provides more immediate insights. It certainly seems better than the alternative of relying on simulations and thought experiments to discern the best diagnostics of research replicability, the approach that now prevails.

Our study uses the most comprehensive dataset related to predictors of replicable research, as we capture relevant data from each of the following sources: Reproducibility Project: Psychology (RPP) [17], the Social Sciences Replication Project (SSRP) [21], the Many Labs Project [22–24], Registered Replication Reports (RRR) [25], and Replications of Important Results in Social Psychology (JSP) [26]. Table 1 and S2 Table present relevant information for each of these projects and the ways that each one operationalized replication success. We also captured data from Curate Science [27], a website that stores individual replication efforts published in psychology’s peer-reviewed journals, and retrieved all applicable peer-reviewed psychological research that appeared on Curate Science. Three hundred and sixteen psychological effects were initially retrieved. Because 6 studies sought to replicate a null effect, they were excluded from the analyses reported here. This decision was based on the consideration that including these studies could potentially introduce bias into our results. Specifically, these studies test for the absence rather than the presence of an effect. They often operate under different statistical assumptions and power considerations. By excluding these studies, we aim to ensure that our findings more accurately reflect the dynamics of replicating substantive psychological effects.

**Table 1:** Replication projects and their measures of replicability.

Project	No. of psychological effects	Original article year	P-value < 0.05 or not (binary)	Percentage of studies replicating the original effect (continuous)	Meta-analysis results significant or not (binary)	The Bayesian analysis results replicating the effect or not? (binary)	Original effect within the confidence interval of replication study or not? (binary)	Small-telescopes Approach (binary)
RPP	96	2008	√				√	
SSRP	21	2010–2015	√		√	√	√	√
Many Labs	57	1935–2014		√	√			
RRR	16	1979–2014	√		√			
JSP	27	1951–2012	√			√		
Individual efforts	93	1971–2013	√		√	√		

Additionally, we were unable to retrieve sample size data for 3 studies, and 26 studies either did not report effect size or did not report sufficient statistics for us to calculate an effect size. A particular study was included in analysis when relevant data were available. In total, 307 studies were included in our analyses for sample size and 284 in our analysis of effect size (due to the absence of data needed to calculate effect size). All data and codes to reproduce our findings are publicly available at the Open Science Framework, https://osf.io/5nvrt/?view_only=2e0369997ba84b61a47241c155996d6a.

We captured data related to the sample size of the original study, and, critically, we defined it as the number of participants in the specific conditions that were subjected to the later replication. If there was a replication study that sought to replicate only some of the conditions included in the original study, we used the reduced sample size corresponding to the sample size of the re-tested conditions. In some cases, this had to be calculated based on the total sample size and the number of conditions. It is important to highlight that the sample sizes in the replication studies may not necessarily correspond to the sample sizes in the original studies. In our analysis, we used information from the original studies as the predictors.

We defined effect size in terms of Cohen’s d of the original effect: the mean difference between the key treatment and control conditions, divided by the standard deviation. Alternative ways of operationalizing effect size, such as R squared, yielded similar results (see Supporting Information). We performed statistical transformations where necessary so that all applicable effect sizes were estimated in Cohen’s d units [28, 29]. We also assessed whether the original study featured a between-subjects design or a within-subjects design, as within-subjects studies typically require smaller sample sizes to achieve the same level of statistical power.

All replication attempts offered a binary indicator of whether the original effect was deemed replicated. For the RPP and SSRP projects, that was typically defined as whether the key null-hypothesis-test yielded a *p* value below the 0.05 threshold. For the Many Labs project, which subjected each effect to multiple replications, success was defined based on whether the null hypothesis test returned a significant result (*p* < 0.05) when results were meta-analyzed across all replication attempts. We used this binary variable as our key outcome variable. Although a binary definition of replication success has been criticized, it is the greatest common denominator of replication success across diverse psychology disciplines.

Using alternative, more nuanced definitions of replication success, such as the percentage of studies replicated in meta-analyses and indices derived from Bayesian analysis, yielded similar patterns. First, we defined replication success as whether meta-analysis returned a significant result, available for 110 studies. Analyses showed that, consistent with results from the main text, sample size did not correlate with meta-analysis replication success, and if anything, the association was negative (*r*_s_ = -0.14, *p* = 0.150) while effect size did positively correlate with replication success (*r*_s_ = 0.35, *p* = .0003). Second, we defined replication success as whether the replication study passed the Bayesian analysis, available for 54 studies. Results showed that again, effect size predicted replicability (*r*_s_ = 0.27, *p* = 0.057). Sample size did not, and as with the meta-analysis analysis, if anything, the association was negative (*r*_s_ = -0.23, *p* = 0.119). In addition, analyses defining replication success as whether the original study’s effect fell within the 95% CI (Confidence Interval) of the replication study’s effect and using the Small Telescopes approach found similar patterns and were reported in the Supporting Information.

## Results

Among all replication projects in psychology, an average of 36.7% of effects were replicated, a figure that should be viewed in the context of the non-representative nature of the studies subjected to replication. We first calculated the two-tailed Spearman’s rank-order correlations, consistent with the previous literature [17]. Across all psychological effects, sample size did not correlate with replication success (*r*_s_ = -0.02, *p* = 0.741). Some researchers [2] have suggested a cell size larger than 20 as a heuristic for determining whether a given study has “enough power.” We calculated cell size for between-subjects studies by dividing their total sample size by the number of key conditions. We then empirically tested this cutoff and found that dichotomizing average cell size into n = < 20 versus n > 20 did not predict replicability (see S4 Table).

**Fig 2:**
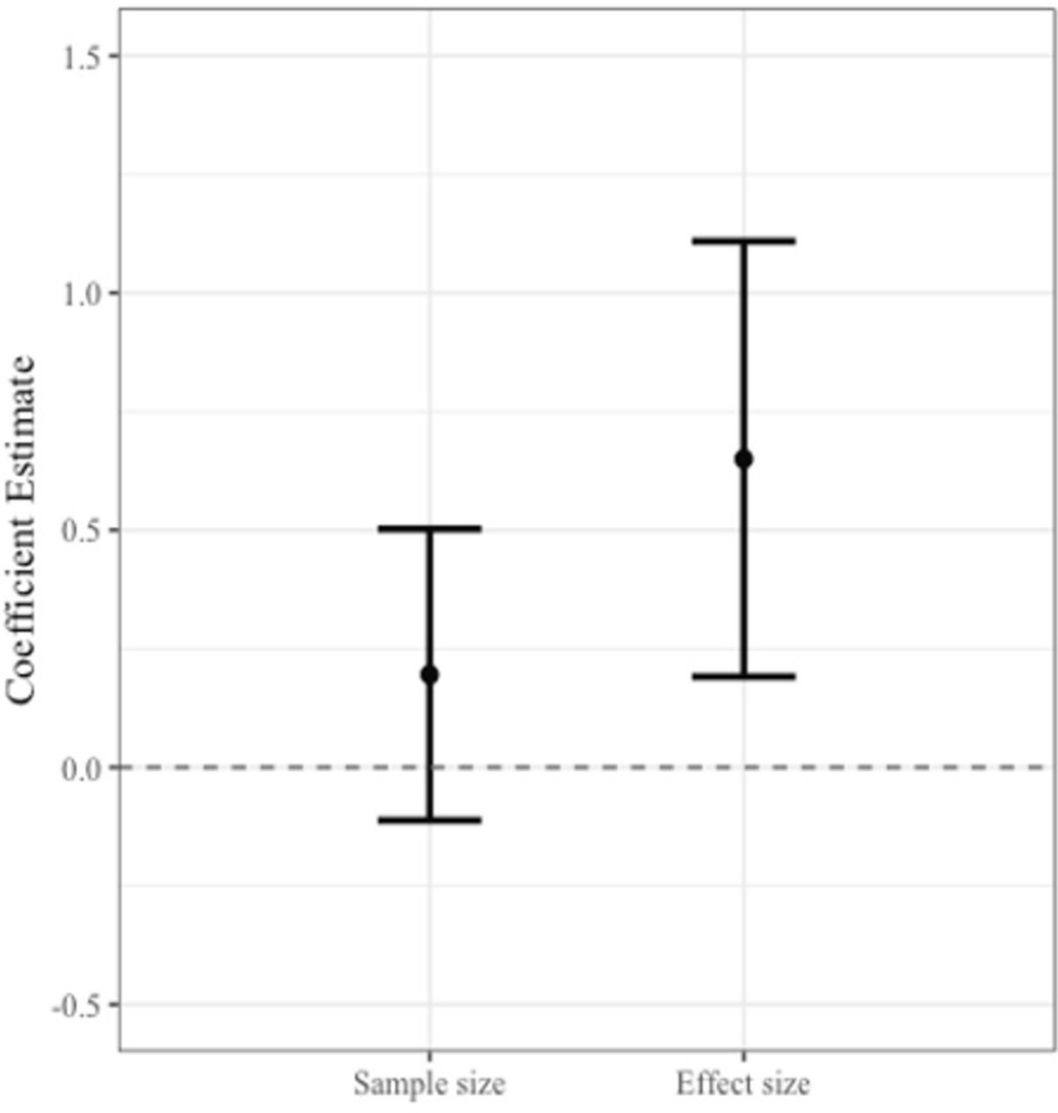
Comparisons of predictors of replicability. Coefficient estimates from separate logistic regressions predicting replication success from the sample size and effect size of the original study, controlling for study design. Error bars represent 95% confidence intervals.

By contrast, effect size positively predicted replication success (see Fig 2 and S3 Table), such that studies with larger effect sizes were more likely to be replicated (*r*_*s*_ = 0.21, *p* < 0.001).

Next, we used logistic regression, controlling for an obvious and pre-specified confound, the use of within vs. between-subjects design (with within-subjects studies coded as -1, between-subjects studies as +1), as within-subjects studies require smaller N to achieve similar power. Due to the small number of correlational studies (only 14), which focused on non-causal relationships and were insufficient for a separate analysis, they were excluded from the main analyses; however, including them yielded the same pattern (see Supporting Information). We report unstandardized regression coefficients. We have chosen to report unstandardized beta coefficients given the diversity of scales among our predictors, which include both dummy variables and continuous variables such as sample size and effect size. This approach enhances interpretability, as it clearly demonstrates the tangible impact of one unit change in any predictor on the likelihood of study replication, which is particularly crucial for our mixed-scale dataset. Because of skew, we log-transformed sample size and effect size, and then entered each one into separate logistic regressions that controlled for study design (within-subjects studies proved relatively more replicable, B = -0.84, SE = 0.19, *p* < 0.0001). Fig 2 displays the coefficients derived from regression models predicting replication success from sample size vs. effect size. Once again, sample size did not predict replicability (B = 0.20, SE = 0.16, *p* = 0.214) but effect size did (B = 0.64, SE = 0.23, *p* = 0.006). There were no interactions of either sample size or effect size with study design, suggesting that these associations were relatively consistent across study design (B = 0.29, SE = 0.21, *p* = 0.159 and B = 0.03, SE = 0.26, *p* = 0.906, respectively).

**Fig 3:**
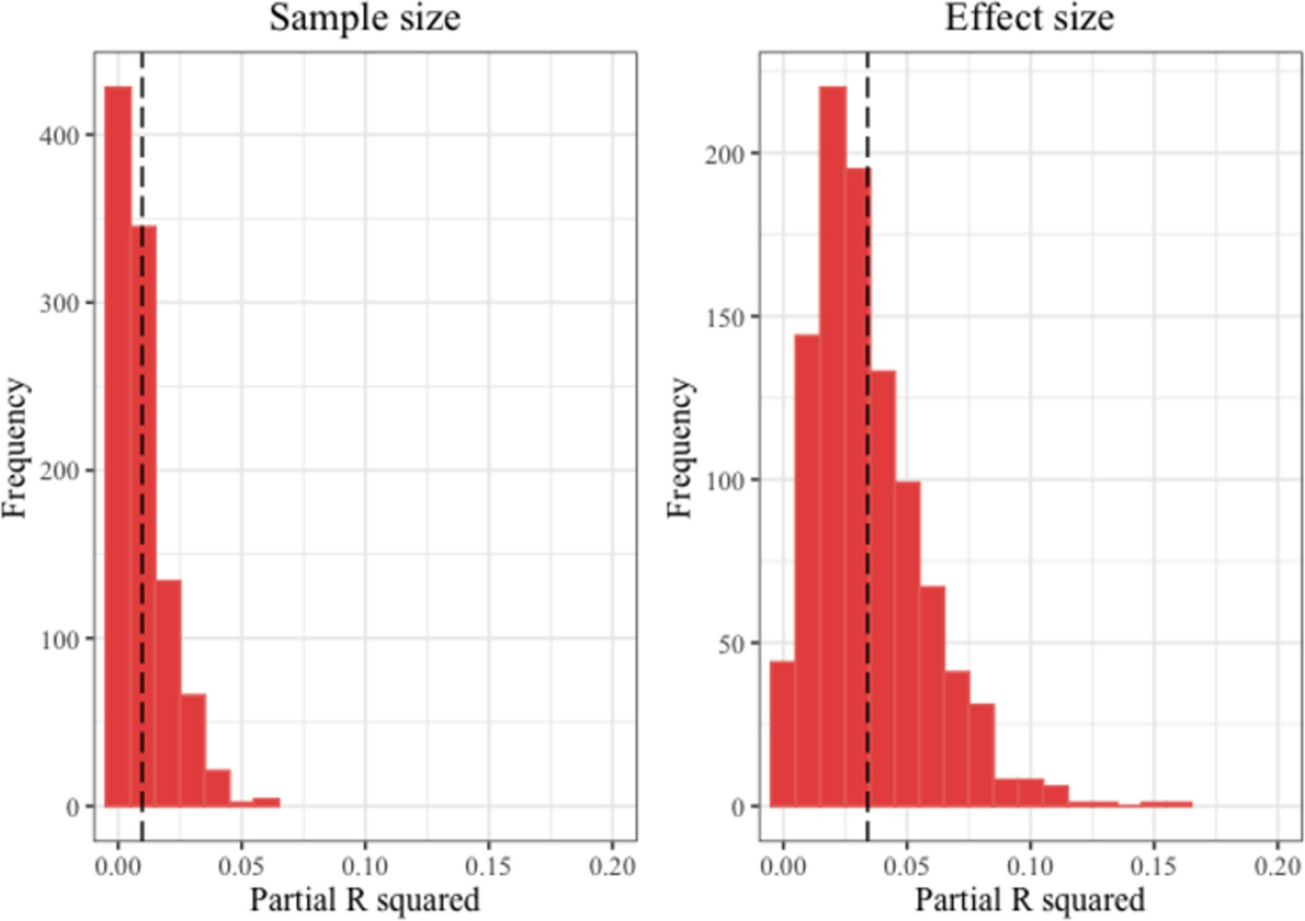
Distribution of bootstrap simulations for replicability predictions by sample and effect sizes. The bootstrap distributions of partial R^2^ for replicability, with dashed vertical lines representing the median partial R^2^ values: 0.66% for sample size and 2.88% for effect size.

To assess the robustness of these findings, we analyzed with 1000 bootstrap simulations of two logistic regression models, one with sample size as a predictor of replicability, and the other with effect size, each controlling for study design (see Fig 3). Relative to the simple model with study design as the sole predictor, adding sample size explained only 0.66% more of the variance in replicability while adding effect size to the model explained 2.88% more variance.

The importance of sample size may depend on the effect size. Larger samples are needed to achieve statistical power for smaller effects. However, there was no interaction between sample size and effect size (both log-transformed) in predicting replication success, B = 0.24, SE = 0.21, *p* = 0.255. That is, for any given effect size, sample size still did not predict replicability. Conversely, for any given sample size, including small ones, effect size did predict replicability [10].

**Fig 4:**
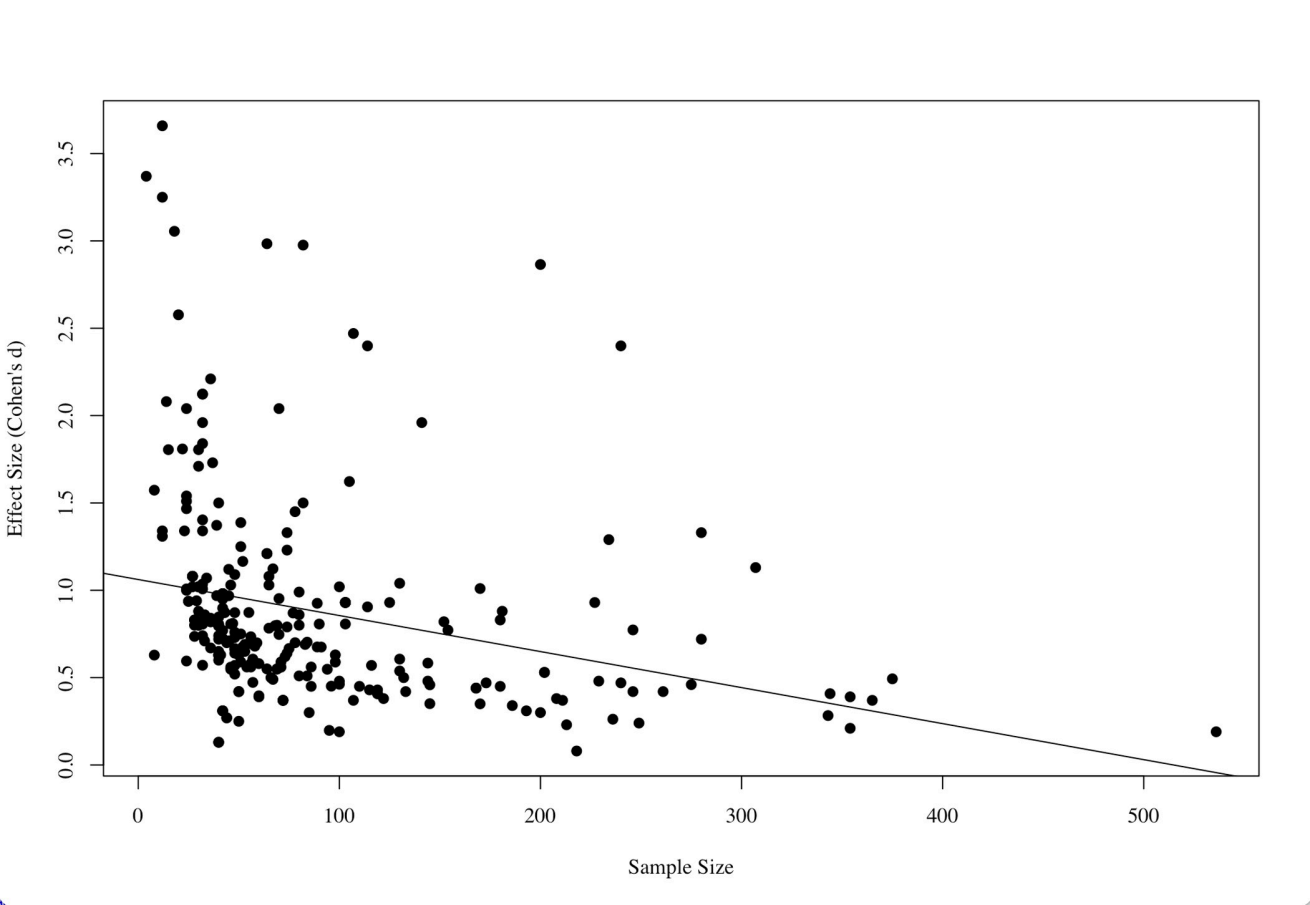
Revealing the trade-off: Larger sample sizes associated with smaller effect sizes. In the actual world, larger samples studies investigated smaller effects (*r*_s_ = -0.49, *p* < 0.001). Five extreme values with N > 1000 and/or Cohen’s d > 4 were not shown in this graph but included in analyses. Excluding them yielded a similar pattern.

Closer inspection reveals that the limited predictive power of sample size may arise from a suppressor effect [30]. Large N studies investigated smaller effects (rs = -0.49, *p* < 0.001; Fig 4), a relationship uncovered in our analysis post-hoc. This pattern was replicated even when we examined individual replication projects like the RPP and Many Labs projects (see S1 Fig). Thus, any gains in power from large N may have been offset by reductions in the investigated effect size. To test the possibility of a suppression effect, both sample size and effect size were entered simultaneously in logistic regression. Consistent with a suppression effect, sample size, as well as effect size, now significantly predicted replicability (B = 0.56, SE = 0.20, *p* = 0.005 and B = 1.03, SE = 0.28, *p* = 0.0002, respectively). Larger sample sizes, it seems, would be predictive of more replicability if their impact were not being suppressed by their association with smaller effect sizes. Statistically lesioning out the influence of effect size from the sample size variable increases the sample size’s predictive power.

Alternative model specifications with different controls (e.g. online vs. lab, social vs. cognitive psychology studies etc.) yielded similar results, and within each replication project, all patterns reported above were replicated (see Supporting Information), a testament to the robustness of the results.

In addition, the Many Labs studies (N = 58) offered a more precise gauge of replicability—the percentage of studies that replicated the original effect. This outcome is less susceptible to sampling and random error that may affect an individual replication’s success and offers a more precise, continuous (0 to 100% studies replicating the original effect) estimate of the robustness of a psychological effect [31]. Using Many Labs data confirmed the same pattern found in our main analysis: sample size could not predict replicability (*r*_s_ = 0.16, *p* = 0.248), while effect size significantly predicted replicability (*r*_s_ = 0.46, *p* = 0.0005), see Supporting Information for details.

While our findings highlight that a larger sample size does not inherently guarantee the replicability of psychological effects, it is important to acknowledge the broader utility of large samples in research. Large-N studies afford more accurate estimations of population parameters, according to the Central Limit Theorem, which states that, given a sufficiently large sample size, the sampling distribution of the mean will be normally distributed, regardless of the distribution of the population. This principle is foundational for inferential statistics, allowing researchers to make more precise confidence interval estimates and hypothesis tests. Moreover, larger sample sizes enhance the ability to detect small but potentially meaningful effects and provide the statistical power necessary to investigate complex interactions and the influence of moderators and mediators. In exploratory research, large samples allow for subgroup analyses that can uncover patterns and effects that may be obscured in smaller samples, thus contributing to the richness and depth of psychological understanding.

Moreover, our findings should be considered in light of potential publication bias [32, 33]. This bias may contribute to the patterns observed, particularly the inverse relationship between sample size and effect size. Publication bias favors studies that achieve statistically significant results—often those with larger effect sizes—which are more likely to be published regardless of sample size. Therefore, the predominance of larger sample sizes in published studies may not reflect their inherent replicability but rather a publication system that privileges certain results, potentially skewing our dataset.

Our data show that, in the best available dataset for assessing the issue, large sample size does not predict replicability, but effect size does. The results suggest that it would be a mistake for editors, reviewers, and funders to use sample size as a heuristic for gauging a research study’s replicability. Indeed, if a heuristic were to be used, effect size would be more merited by the empirical evidence. To be clear, for any specific study, increasing sample size will, by necessity, lead to greater replicability, because larger samples yield more power and thus a greater likelihood of identifying a true effect—*when holding the given study’s effect size (and other attributes) constant*. The key point is that in a pool of scientific studies, larger sample sizes are associated with different studies: in particular, studies that investigate smaller effect sizes (*r*_s_ = -0.49, *p* < 0.001).

### Survey study

A research question that naturally follows these findings is: To what extent do psychological scientists espouse large sample size as a heuristic for judging a study’s replicability?

**Fig 5:**
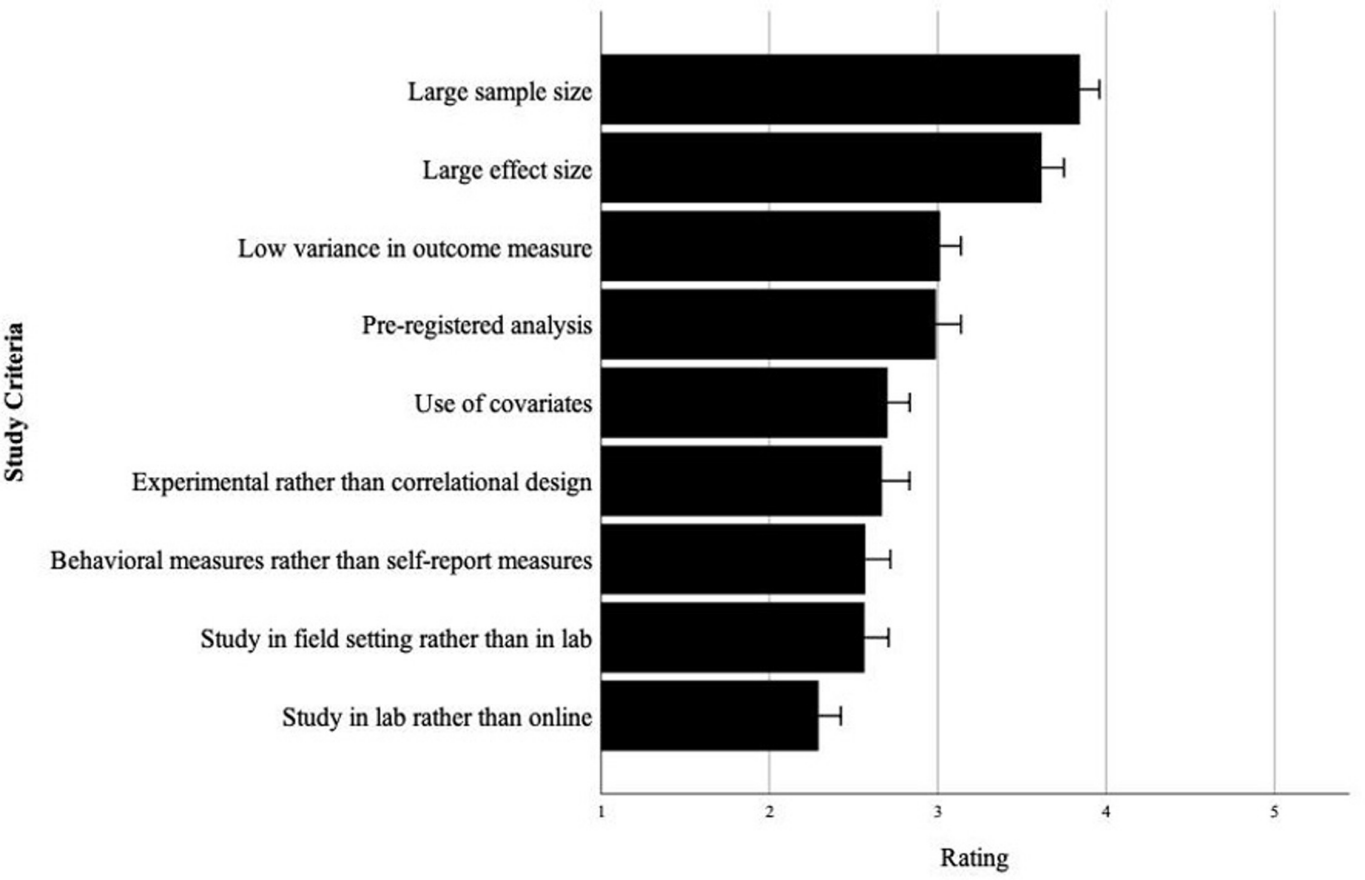
Perceived importance of study criteria for replicability among psychological scientists. Psychological scientists rated “large sample size” as the most important criterion in predicting the replicability of a study. Responses were asked “in judging a research study, to what extent do you think each of the following factors would predict its replicability?” and provided on a 5-point scale ranging from “not at all” to “an extreme amount.” Error bars represent ±1 SE.

To address this question, we surveyed psychological scientists, broadly defined, by sending out invitation links to a survey about “replicability in psychological research” through social media platforms and the Society for Personality and Social Psychology listserv. Similar assessment procedures have been used to study the judgments of psychological scientists. Ten undergraduate students replied to the survey and were excluded from all analyses, resulting in a final sample size of 215 psychological scientists. Sixty-nine percent had served as a reviewer for a psychology journal, 20% as an editor. Ninety percent had a Ph.D. or were pursuing one. Respondents first read that “there are many factors in a research study that might predict its replicability (i.e., the likelihood of replicating in future studies)” and were then asked, “in judging a research study, to what extent do you think each of the following factors would predict its replicability?” Respondents rated each of nine criteria (see Fig 5, which also displays the average importance rating ascribed to each study criterion).

Among all participants, fifty-five percent had earned a Ph.D. in psychology, 35% reported pursuing a Ph.D. degree in psychology, and the remainder either had or were pursuing a Master’s degree in psychology. Confining analysis only to respondents with a Ph.D. in psychology yielded the same results (see Supporting Information). We randomized the order of presentation of nine study criteria between participants, and for each one, they rated the extent to which they thought it predicted a study’s replicability on a 5-point scale, with higher numbers signifying greater importance.

Respondents rated “large sample size” as the most important criterion (M = 3.84), judging it to be significantly more important than the second most important factor, “large effect size” (M = 3.61), t(214) = 3.21, *p* = 0.002. Interestingly, they saw large sample size as more important than another heavily advocated research practice, pre-registered analysis (M = 2.97), t(214) = 10.49, *p* < 0.001. When we looked specifically at scientists who had served as reviewers and editors, the pattern proved stronger: The tendency to rate sample size as a more important criterion (relative to all other criteria’s importance rating averaged) was stronger among reviewers and editors than among the remainder of our sample of psychological scientists (see S1 Table). These data provide evidence for the N-Heuristic among scientific researchers—a reflection of the prevailing doctrine that N is a key heuristic in judging the replicability of scientific research.

In a sense, the use of sample size as a key heuristic in judging a research study’s replicability assumes a simple and mathematically deducible world—as often represented in data simulations. In this world, as N increases, the investigated effect size remains the same. Table 2 illustrates this ideal world and compares it with the actual world empirically documented in our research. In the ideal world, N is a good gauge of power (and thus replicability) because all other factors such as effect size are held constant. But in the real world, N is not a good gauge, because larger studies are associated with smaller effect sizes.

**Table 2:** Comparison of manuscripts in ideal vs. actual world scenarios based on power analysis. Manuscripts in the ’Ideal World’ column represent expected values based on the power calculated with G*Power, while the ’Actual World’ column reflects observed data from our meta-analysis. Highlighted rows indicate the most highly powered research papers.

	Ideal World				Actual World		
	N	Effect Size	Power (%)		N	Effect Size	Power (%)
Manuscript 1	20	0.3	26	Manuscript 1	12	1.3	53
Manuscript 2	30	0.3	38	Manuscript 2	30	0.9	66
Manuscript 3	50	0.3	59	Manuscript 3	50	0.6	55
Manuscript 4	80	0.3	79	Manuscript 4	80	0.5	60
Manuscript 5	100	0.3	88	Manuscript 5	100	0.5	70
…	…	…	…	…	…	…	…
Manuscript 307	200	0.3	99	Manuscript 307	193	0.31	57

These results show that if reviewers and editors increasingly come to rely on large N as a heuristic or peripheral-route cue in judging the quality of research [32, 33], they may be no different from other decision-makers faced with a complex judgmental task under time constraints, cognitive load, a busy schedule, and so on. Small studies are viewed with suspicion. But we should consider the possibility that a small study revealing a big effect may be just as replicable and important as a big study with a small effect. In fact, our data suggest that the former is more replicable than the latter. Moreover, a sequential (and pre-registered) series of small studies with big effects would still be more trustworthy than a single big study showing a small effect.

## Discussion

An important question at the heart of our research is, “What diagnostics predict replicable research?” In psychological science, N has been seized on as a heuristic for making this assessment, to the detriment of our field. Of course, all else equal, larger N is better. But all else is not equal, as we find. Large N studies investigate smaller effect sizes, which are less replicable. Put differently, small-sample studies with big effects replicate more often than large-sample studies with small effects.

Obviously, correlation is not causation, and thus, it is worth reiterating that our goal is a diagnostic not a causal one—a kind of epidemiology of science. There are many reasons to assume that small N studies will be less replicable—the problem “spot” in psychological science. Yet they are not. A better diagnostic for poor replicability is a small effect size. To illustrate our contribution concretely, imagine two papers submitted to a journal, each addressing the same hypothesis. The first paper reports two tightly controlled studies conducted in the lab. Because of practical constraints, the sample size is small (N = 15 per condition), but the effect size is consistently large. The second paper reports two M-Turk studies featuring over 1000 participants each, with a consistently small effect size. Current norms favor the publication of the second paper, not the first. But our results suggest that the first paper would be a better bet. At the very least, a bias against the small N study—which our survey study suggests investigators will have—does not appear to be warranted by the empirical evidence to date in the data we have available.

Of course, many factors may obscure the relationship between N and replicability. There is much variety in the studies subjected to replication attempts, though we tried to control for the most obvious confounds (e.g., the use of within- versus between-subjects designs, and the specific subfield of psychology). But this is precisely our point: In the welter of the real world—with the variety of attributes of a research study—sample size is not a sufficiently strong enough predictor to rise above various sources of noise [8, 9]. Critically, as we also show, this does not mean it is impossible to find a useful diagnostic: Another largely unheralded research attribute—effect size—is revealed to be robustly predictive of replicability. However, one key challenge is the potential inflation of effect sizes due to the reduced precision inherent in small N studies. This limitation can lead to overestimations of effect magnitude, which may not be replicable in studies with larger and more diverse sample groups.

There are several limitations to our findings. First, the studies subjected to replication and our surveyed respondents were not representative samples. Thus, further attempts to assess the generality of our findings are needed. Second, we do not, by necessity, know the *causal* impact of policies that encourage large N or effect sizes. Our results do *not* suggest that large sample sizes are unimportant. While larger sample sizes do improve the precision and generalizability of the effects observed, they also tend to dilute the magnitude of effect sizes, reflecting more modest but potentially more widely applicable effects. Large sample size increases statistical power, and they should be encouraged and incentivized. Instead, our data call for a more comprehensive consideration of issues beyond large N.

While large N is a pathway to achieve statistical power, we also think that more attention could be given to the other paths to statistical power. Researchers who choose to—or have to—use small samples have several means for increasing power. They can conduct studies in the lab where they can better control for extraneous variables by ensuring a constant situation for all participants; they can increase the psychological impact of their manipulation by using an immersive “event” manipulation; they can use baseline measures and pre-specified covariates; where feasible, they can use within-subjects designs; and they can increase the reliability of their measures [11, 32].

In light of our data, editors and reviewers can reasonably entertain the possibility that a small-sample study can sometimes be well-powered and likely to be replicable. They might also consider the utility of weighting effect size more heavily in publication decisions. On the other hand, any rigid heuristic should be avoided, as it can become a stifling basis for exclusion.

## Supporting information

S1 Fig Sample size reversely correlated with effect size for studies in the RPP project (left panel) and in the Many Labs project (right panel).
(TIF)

S1 Table Mean rating of nine study criteria in predicting study replicability split by participant demographics. (DOCX)

S2 Table Replication projects characteristics. (DOCX)

S3 Table Spearman correlation matrix. (DOCX)

S4 Table Descriptive analysis of replication success rate at different levels of P-value for the original study. (DOCX)

## References

[1] Nosek BA, Ebersole CR, DeHaven AC, Mellor DT. The preregistration revolution. Proc Natl Acad Sci. 2018 Mar 12;115(11):2600–6. doi: 10.1073/pnas.1708274114 29531091 PMC5856500

[2] Simmons JP, Nelson LD, Simonsohn U. False-positive psychology: Undisclosed flexibility in data collection and analysis allows presenting anything as significant. Psychol Sci. 2011 Oct 17;22(11):1359–66. doi: 10.1177/0956797611417632 22006061

[3] Maxwell SE, Lau MY, Howard GS. Is psychology suffering from a replication crisis? What does “failure to replicate” really mean? Am Psychol. 2015;70(6):487–98. doi: 10.1037/a0039400 26348332

[4] Cooper ML. Editorial. J Pers Soc Psychol. 2016;110:431–4. doi: 10.1037/pspp0000033 26963765

[5] Kawakami K. Editorial. J Pers Soc Psychol. 2015;108:58–9. doi: 10.1037/pspi0000013 25603368

[6] Kitayama S. Editorial. J Pers Soc Psychol. 2017;112:357–60. doi: 10.1037/pspi0000088 28221091

[7] Nature Human Behaviour. How we evaluate your manuscripts. 2021 Apr 28. 10.1038/s41562-019-0778-0 31723272

[8] Ioannidis JP. Why most published research findings are false. PLoS Med. 2005;2:e124. doi: 10.1371/journal.pmed.0020124 16060722 PMC1182327

[9] Fraley RC, Vazire S. The N-pact factor: Evaluating the quality of empirical journals with respect to sample size and statistical power. PLoS One. 2014;9(10):e109019. doi: 10.1371/journal.pone.0109019 25296159 PMC4189949

[10] Munafò MR, et al. A manifesto for reproducible science. Nat Hum Behav. 2017;1:1–9. doi: 10.1038/s41562-016-0021 33954258 PMC7610724

[11] Aronson E, Ellsworth PC, Carlsmith JM, Gonzalez MH. Methods of research in social psychology. New York: McGraw-Hill Humanities/Social Sciences/Languages; 1990.

[12] Anderson CA, et al. The MTurkification of social and personality psychology. Pers Soc Psychol Bull. 2019;45:842–50. doi: 10.1177/0146167218798821 30317918

[13] Forstmeier W, Wagenmakers E, Parker TH. Detecting and avoiding likely false-positive findings–a practical guide. Biol Rev. 2017;92:1941–68. doi: 10.1111/brv.12315 27879038

[14] Sassenberg K, Ditrich L. Research in social psychology changed between 2011 and 2016: Larger sample sizes, more self-report measures, and more online studies. Adv Methods Pract Psychol Sci. 2019;2:107–14. doi: 10.1177/2515245919838781

[15] Giner-Sorolla R. From crisis of evidence to a “crisis” of relevance? Incentive-based answers for social psychology’s perennial relevance worries. Eur Rev Soc Psychol. 2019;30:1–38. doi: 10.1080/10463283.2019.1622781

[16] Berkman ET, Wilson SM. So useful as a good theory? The practicality crisis in (social) psychological theory. Perspect Psychol Sci. 2021;16:864–74. doi: 10.1177/1745691620969650 33412079 PMC8260606

[17] Open Science Collaboration. Estimating the reproducibility of psychological science. Science. 2015;349:aac4716. doi: 10.1126/science.aac4716 26315443

[18] Yang Y, Youyou W, Uzzi B. Estimating the deep replicability of scientific findings using human and artificial intelligence. Proc Natl Acad Sci. 2020;117:10762–8. doi: 10.1073/pnas.1909046117 32366645 PMC7245108

[19] Altmejd A, et al. Predicting the replicability of social science lab experiments. PLoS One. 2019;14:e0225826. doi: 10.1371/journal.pone.0225826 31805105 PMC6894796

[20] Stanley TD, Carter EC, Doucouliagos H. What meta-analyses reveal about the replicability of psychological research. Psychol Bull. 2018;144:1325. doi: 10.1037/bul0000169 30321017

[21] Camerer CF, et al. Evaluating the replicability of social science experiments in Nature and Science between 2010 and 2015. Nat Hum Behav. 2018;2:637–44. doi: 10.1038/s41562-018-0399-z 31346273

[22] Klein R, et al. Investigating variation in replicability: A “many labs” replication project. Open Science Framework. 2014. [No DOI available].

[23] Ebersole CR, et al. Many Labs 3: Evaluating participant pool quality across the academic semester via replication. J Exp Soc Psychol. 2016;67:68–82. doi: 10.1016/j.jesp.2015.10.012

[24] Klein RA, et al. Many Labs 2: Investigating variation in replicability across samples and settings. Adv Methods Pract Psychol Sci. 2018;1:443–90. doi: 10.1177/2515245918810225

[25] Simons DJ, Holcombe AO. Registered replication reports. APS Observer. 2014;27. 10.1177/1745691614543974 26186757

[26] Nosek BA, Lakens D. Replications of important results in social psychology. Soc Psychol (Gott). 2014;45.

[27] Aarts E, LeBel E. Curate Science: A platform to gauge the replicability of psychological science. 2016

[28] Cohen J. The effect size. Statistical Power Analysis for the Behavioral Sciences. 1988:77–83.

[29] Rosenthal R, Cooper H, Hedges L. Parametric measures of effect size. In: The Handbook of Research Synthesis. 1994:621, 231–44.

[30] Friedman L, Wall M. Graphical Views of Suppression and Multicollinearity in Multiple Linear Regression. The American Statistician. 2005;59(2):127–136. doi: 10.1198/000313005X41337

[31] Van Bavel JJ, Mende-Siedlecki P, Brady WJ, Reinero DA. Contextual sensitivity in scientific reproducibility. Proc Natl Acad Sci U S A. 2016;113(23):6454–6459. doi: 10.1073/pnas.1521897113 27217556 PMC4988618

[32] Fanelli D, Costas R, Ioannidis JPA. Meta-assessment of bias in science. Proc Natl Acad Sci U S A. 2017;114(14):3714–3719. doi: 10.1073/pnas.1618569114 28320937 PMC5389310

[33] Kühberger A, Fritz A, Scherndl T. Publication bias in psychology: A diagnosis based on the correlation between effect size and sample size. PLoS One. 2014;9(9):e105825. doi: 10.1371/journal.pone.0105825 25192357 PMC4156299

