# Supplementary figures and images for: Challenging the N-Heuristic: Effect size, not sample size, predicts the replicability of psychological science
Source: PLoS One. 2024 Aug 23;19(8):e0306911. doi: 10.1371/journal.pone.0306911 (PMC11343368; doi:10.1371/journal.pone.0306911)

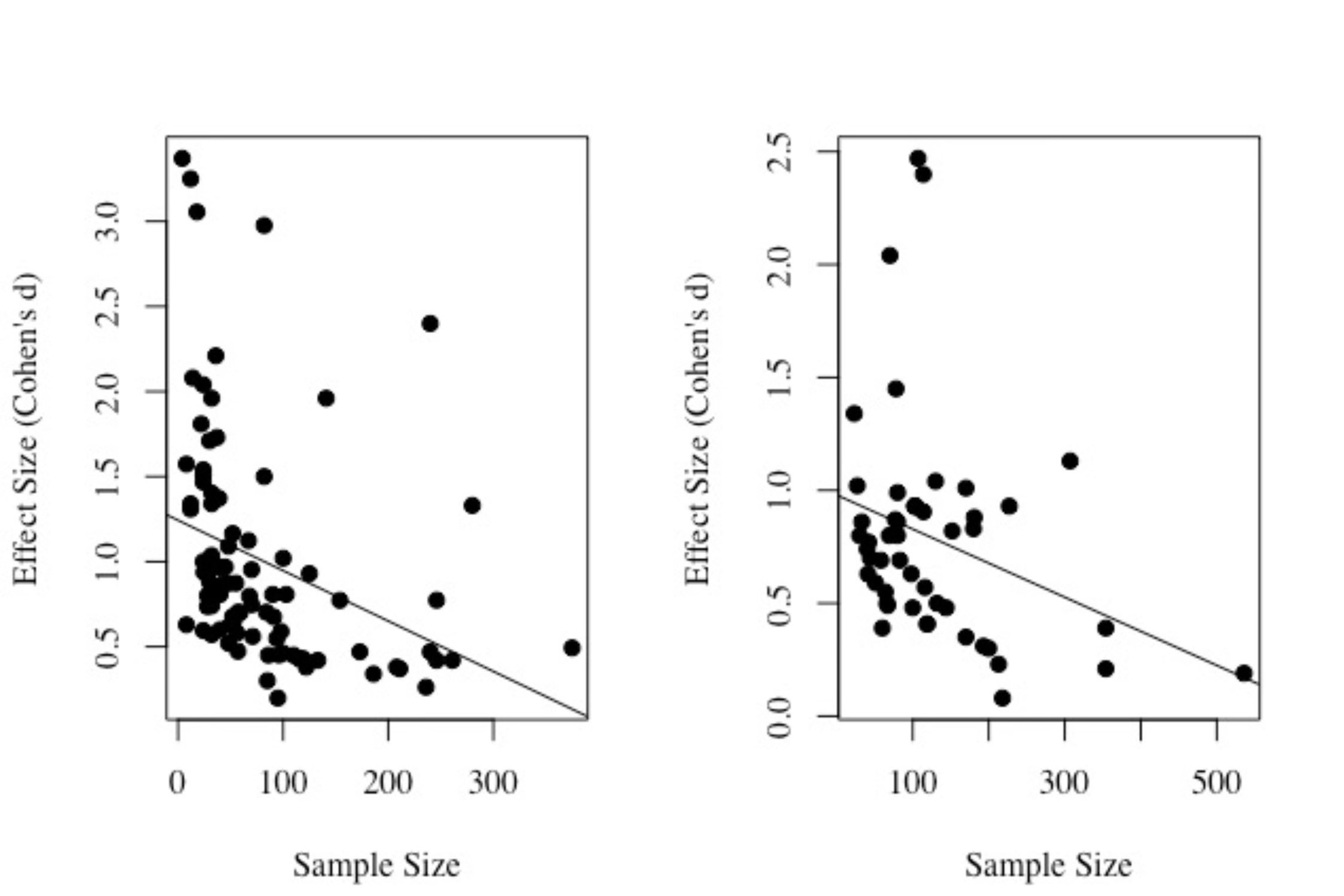

Supplement: S1 Fig — Sample size reversely correlated with effect size for studies in the RPP project (left panel) and in the Many Labs project (right panel). (TIF) [file pone.0306911.s001.tif]
