# Supplementary material for: Challenging the N-Heuristic: Effect size, not sample size, predicts the replicability of psychological science
Source: PLoS One. 2024 Aug 23;19(8):e0306911. doi: 10.1371/journal.pone.0306911 (PMC11343368; doi:10.1371/journal.pone.0306911)
Supplement: S1 Table — (DOCX) [file pone.0306911.s002.docx]

Table S1.

Mean rating of nine study criteria in predicting study replicability split by participant demographics.

| Mean Rating of  Study Criteria | Have a Ph.D. degree? | | Served as a psychology journal reviewer? | | Served as a psychology journal editor? | |
| --- | --- | --- | --- | --- | --- | --- |
|  | Yes | No | Yes | No | Yes | No |
| N | 119 | 96 | 149 | 66 | 44 | 171 |
| Large sample size | 3.89 | 3.77 | 3.95 | 3.59 | 3.98 | 3.80 |
| Large effect size | 3.66 | 3.55 | 3.66 | 3.48 | 3.57 | 3.62 |
| Pre-registered analysis | 2.82 | 3.17 | 2.87 | 3.20 | 2.68 | 3.05 |
| Behavioral measures rather than self-report measures | 2.34 | 2.86 | 2.39 | 2.98 | 2.30 | 2.64 |
| Experimental rather than correlational design | 2.41 | 2.98 | 2.47 | 3.11 | 2.30 | 2.76 |
| Study in lab rather than online | 2.17 | 2.46 | 2.21 | 2.50 | 2.11 | 2.35 |
| Study in field setting rather than in lab | 2.42 | 2.79 | 2.51 | 2.76 | 2.32 | 2.65 |
| Use of covariates | 2.58 | 2.83 | 2.59 | 2.94 | 2.55 | 2.74 |
| Low variance in outcome measures | 3.01 | 3.05 | 3.00 | 3.09 | 3.07 | 3.02 |
