# Supplementary material for: Challenging the N-Heuristic: Effect size, not sample size, predicts the replicability of psychological science
Source: PLoS One. 2024 Aug 23;19(8):e0306911. doi: 10.1371/journal.pone.0306911 (PMC11343368; doi:10.1371/journal.pone.0306911)
Supplement: S2 Table — (DOCX) [file pone.0306911.s003.docx]

Table S2.

Replication projects characteristics.

| Project | No. of psychological effects | Median sample size | Median effect size (Cohen’s d) | Percentage (%) of effects replicated |
| --- | --- | --- | --- | --- |
| RPP | 96 | 51 | 0.81 | 37.5 |
| SSRP | 21 | 54 | 0.97 | 61.9 |
| Many Labs | 57 | 103 | 0.72 | 57.9 |
| RRR | 16 | 51 | 0.68 | 18.8 |
| JSP | 27 | 47 | 0.76 | 25.9 |
| Individual efforts | 93 | 42 | 0.71 | 23.7 |
| Total | 310 | 53 | 0.76 | 36.8 |
