# Supplementary material for: Challenging the N-Heuristic: Effect size, not sample size, predicts the replicability of psychological science
Source: PLoS One. 2024 Aug 23;19(8):e0306911. doi: 10.1371/journal.pone.0306911 (PMC11343368; doi:10.1371/journal.pone.0306911)
Supplement: S3 Table — (DOCX) [file pone.0306911.s004.docx]

Table S3.

Spearman correlation matrix. P-value in parentheses. *** represents correlation is significant at the 0.001 level (2-tailed).

|  | Sample Size | Effect Size  (Cohen’s d) | Replicability |
| --- | --- | --- | --- |
| Sample Size | 1 |  |  |
| Effect Size  (Cohen’s d) | -0.49***  (< 0.001) | 1 |  |
| Replicability | -0.02  (0.741) | 0.21***  (< 0.001) | 1 |
