# Supplementary material for: Challenging the N-Heuristic: Effect size, not sample size, predicts the replicability of psychological science
Source: PLoS One. 2024 Aug 23;19(8):e0306911. doi: 10.1371/journal.pone.0306911 (PMC11343368; doi:10.1371/journal.pone.0306911)
Supplement: S4 Table — (DOCX) [file pone.0306911.s005.docx]

**Table S4.**

Descriptive analysis of replication success rate at different levels of P-value for the original study.

|  | P-value cutoff  at *p* = 0.05 | | P-value cutoff  at *p* = 0.005 | | P-value cutoff  at *p* = 0.001 | |
| --- | --- | --- | --- | --- | --- | --- |
|  | No. of studies | Percentage of studies replicated | No. of studies | Percentage of studies replicated | No. of studies | Percentage of studies replicated |
| Original study significant | 277 | 34.3% | 110 | 50.9% | 75 | 61.3% |
| Original study not significant | 6 | 16.7% | 173 | 23.1% | 208 | 24.0% |

**References**

1. Cohen J. The effect size. In: Statistical Power Analysis for the Behavioral Sciences. 1988. p. 77–83.

2. Rosenthal R, Cooper H, Hedges L. Parametric measures of effect size. In: The Handbook of Research Synthesis. 1994. p. 621, 231–44.

3. Hosmer DW, Lemeshow S, Sturdivant RX. Applied logistic regression. 3rd ed. John Wiley & Sons; 2013.

4. Kleinbaum DG, Klein M. Logistic regression: A self-learning text. 3rd ed. Springer; 2010.

5. Tabachnick BG, Fidell LS. Using multivariate statistics. 7th ed. Pearson; 2019.

6. Cumming G, Maillardet R. Confidence intervals and replication: Where will the next mean fall? Psychol Methods. 2006;11(3):217. doi: 10.1037/1082-989X.11.3.217.

7. Ioannidis JPA. The proposal to lower P value thresholds to .005. JAMA. 2018;319(14):1429–30. doi: 10.1001/jama.2018.1536.

8. Benjamin DJ, et al. Redefine statistical significance. Nat Hum Behav. 2018;2(1):6–10. doi: 10.1038/s41562-017-0189-z.
